# Real-time terahertz digital holography with a quantum cascade laser

**DOI:** 10.1038/srep13566

**Published:** 2015-08-28

**Authors:** Massimiliano Locatelli, Marco Ravaro, Saverio Bartalini, Luigi Consolino, Miriam S. Vitiello, Riccardo Cicchi, Francesco Pavone, Paolo De Natale

**Affiliations:** 1 INO-CNR, Istituto Nazionale di Ottica, Largo E. Fermi 6, Firenze, I-50125, Italia; 2 LENS, European Laboratory for NonLinear Spectroscopy, Via N. Carrara 1, Sesto Fiorentino (Firenze), I-50019, Italia; 3 NEST-CNR, Istituto Nanoscienze and Scuola Normale Superiore, Piazza San Silvestro 12, Pisa I-56127, Italia; 4 Dipartimento di Fisica e Astronomia, Università di Firenze, Via Sansone 1, Sesto Fiorentino (Firenze), I-50019, Italia

## Abstract

Coherent imaging in the THz range promises to exploit the peculiar capabilities of these wavelengths to penetrate common materials like plastics, ceramics, paper or clothes with potential breakthroughs in non-destructive inspection and quality control, homeland security and biomedical applications. Up to now, however, THz coherent imaging has been limited by time-consuming raster scanning, point-like detection schemes and by the lack of adequate coherent sources. Here, we demonstrate real-time digital holography (DH) at THz frequencies exploiting the high spectral purity and the mW output power of a quantum cascade laser combined with the high sensitivity and resolution of a microbolometric array. We show that, in a one-shot exposure, phase and amplitude information of whole samples, either in reflection or in transmission, can be recorded. Furthermore, a 200 times reduced sensitivity to mechanical vibrations and a significantly enlarged field of view are observed, as compared to DH in the visible range. These properties of THz DH enable unprecedented holographic recording of real world dynamic scenes.

Coherent imaging is a well-established technique in the THz range^1^,^2^,^3^, since the traditional approach to THz sensing, based on rectified ultra-short pulses, inherently lends itself to coherent detection schemes^1^. However, the reported 2D imaging capabilities of such powerful spectroscopic systems are restrained by the μW level available THz power^4^, while the need of scanning the THz pulses delay line limits the maximum frame rate. In addition, the acquisition apparatus is typically cumbersome and, thus, unsuitable for in-situ analysis. Alternative coherent imaging schemes, based on Quantum Cascade Lasers (QCLs), exploit self-mixing detection^5^,^6^, or heterodyne mixing between QCLs and gas lasers^7^ or between QCLs and frequency combs^8^. Thanks to the compact size, the broad spectral coverage and a continuous-wave output power as high as hundred mW^9^,^10^,^11^, THz QCLs are widespread sources for imaging applications and, thanks to sub-kHz intrinsic line widths^12^,^13^, they are well suited for coherent imaging. However, whereas the high QCL power has enabled real-time incoherent imaging based on arrays of direct detectors^14^,^15^, the above mentioned coherent imaging schemes are inherently slow, as they all require the sample to be scanned through the focused THz beam.

An elegant approach to single-shot acquisition of amplitude and phase THz images, using QCLs and micro-bolometer focal plane arrays, is represented by Digital Holography^16^. In its so-called off-axis configuration, the acquired holograms result from the interference between the radiation scattered by the target or transmitted through it, and a reference beam directly impinging on the camera array at an angle θ. If the hologram is recorded with a sufficiently wide angle, during its numerical reconstruction the two diffraction orders are completely separated and a clear reconstructed image is obtained. However, due to the finite dimension of the camera pixels, beyond a certain threshold value of θ, interference fringes become too narrow to be correctly sampled. This is a specific drawback of DH with respect to classical holography, which ultimately limits the field of view. Furthermore, as for any holographic technique, DH is strongly perturbed by environmental vibrations and is thus well suited only for imaging of static targets.

The above constraints are particularly restrictive for visible DH, which can only be performed on small targets and in an environment where mechanical vibrations are efficiently damped. Conversely, infrared DH inherently benefits from wavelength scaling of both linear field of view and mechanical stability requirements^17^,^18^: i) the maximum lateral dimension D of a target at a certain distance d is equal to D = λd/d_p_, where d_p_ is the detector pixel pitch and λ is the wavelength; ii) the interferometric pattern containing the wavefront information is erased if the amplitude of environmental vibrations occurring during the acquisition is comparable to the wavelength; a holographic system is, therefore, 200 times more robust against vibrations in the THz range (at e.g. 100 microns) than in the visible range (at e.g. 0.5 microns). In this respect, the recent development of Mid-IR DH systems based on micro-bolometer arrays and CO_2_ lasers^19^ or, more recently, QCLs^20^, allowed acquisition of holographic videos of human size dynamic scenes out of laboratory conditions. Moreover, the possibility to tailor the focusing depth, starting from a single Mid-IR hologram recorded in a lensless configuration, has recently allowed detecting human targets beyond a curtain of smoke and flames^21^. Working at progressively longer wavelengths in the far-infrared is an even more desirable goal, which could further increase DH capabilities. However, despite such potential, THz DH is an almost unexplored technique, mainly due to the limited performances of THz coherent sources and detectors. The most relevant experimental works published to date are based on a frequency multiplied solid state oscillator^22^, a gas laser^23^ and, more recently, a multimode THz QCL^24^, and report proof of principle hologram acquisition of simple static transmission masks. Preliminary results of real-time displacement measurements in the THz range were obtained by means of speckle metrology using the THz radiation of a Free Electron Laser^25^.

## Results and Discussion

In this work, we highlight the potential of THz DH reporting, for the first time, on real-time hologram acquisition and processing of various samples of practical interest. In particular, we show DH at 2.8 THz using a 4 mW QCL and a microbolometric focal plane array. We first demonstrate the possibility to record and reconstruct THz digital holograms of both static and dynamic scattering objects. Secondly, we show amplitude and phase images of transparent samples extracted from THz transmission holograms.

**Figure 1:**
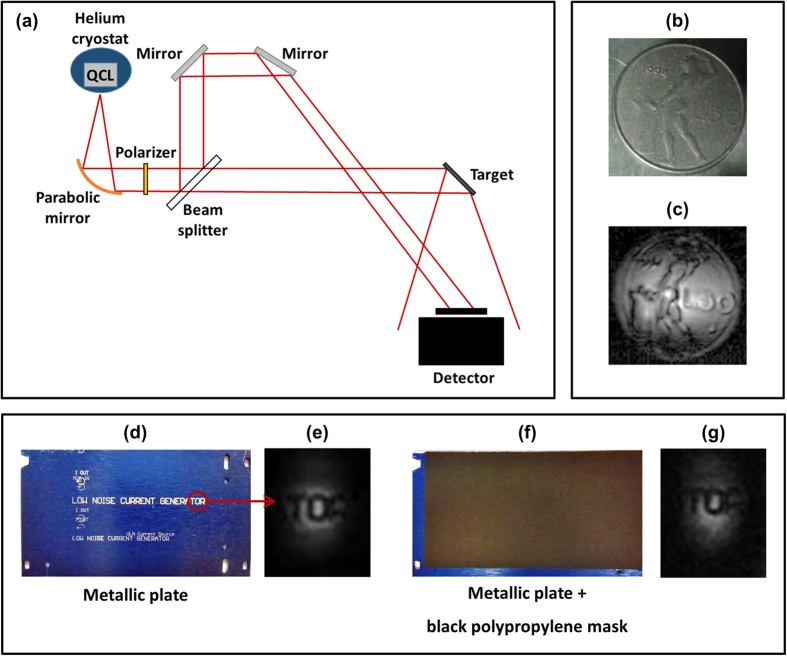
THz DH of scattering targets. (**a**) Sketch of the experimental setup. (**b**) Picture of the imaged 50 Lire coin. (**c**) Reconstruction of the scattered wavefront amplitude. (**d**) Picture of the imaged metallic plate with the inscription “LOW NOISE CURRENT GENERTOR”. **(e)** Wavefront amplitude reconstruction of the final portion of the inscription, imaged while translating the plate at a constant speed of 5 mm/s (see also online material). **(f**) The same metallic plate covered by a 1 mm black polypropylene mask. (**g**) Wavefront amplitude reconstruction of the final portion of the inscription covered by the black mask, imaged while translating the plate at a constant speed of 5 mm/s (see also online material).

Experiments of scattering DH were based on the experimental set-up shown in Fig. 1(a): the laser beam was split into two beams of comparable intensities: the so-called object beam, directed towards the target to be investigated, and the reference beam, directed towards the thermocamera (devoid of objective). The interference pattern obtained by the superposition of the reference beam with the radiation scattered by the target was recorded and numerically processed, in real-time (5 frame/s), in order to obtain the wavefront amplitude reconstruction. Thanks to such real-time capability, we could live adjust both recording and reconstruction parameters (beam intensity balancing, focusing, filtering, etc.) in order to improve the image quality. In a first experiment, a 50 Lire Italian (old) coin, preventively sandblasted to increase the amount of scattered radiation, was employed as a static target (Fig. 1(b)); the wavefront amplitude reconstruction, obtained after various image processing steps (see methods), is shown (Fig. 1(c)). Given the camera pixel pitch, the long wavelength (≈107 μm) results in a field of view of about 130° × 130°. At the chosen recording distance (3.5 cm), this corresponds to an image size of about 15 cm × 15 cm versus a 0.35 cm × 0.35 cm image of a typical digital hologram in the visible range (λ_vis_ = 0.5 μm , d_pvis_ = 5 μm). However, due to mW CW power available from our QCL, such a large area could not be entirely irradiated and only smaller objects could be investigated. A state of the art THz QCL, with output power of about 100 mW, would allow exploiting the full field of view.

In a second experiment, the capability of our system to record and reconstruct real-time scattering holograms of a moving sample was demonstrated. A metallic plate with an etched inscription (Fig. 1(d)) was fixed to a motorized linear stage so that all inscription parts could be irradiated during the plate translation. Taking advantage of the low sensitivity to vibrations we were able to reconstruct such dynamic target hologram (see online video), the quality of the amplitude image being preserved up to a maximum stage speed of 5 mm/s (Fig. 1(e)). This test was successfully repeated after covering the target with a 1 mm thick black polypropylene plate (Fig. 1(f)): the inscription, completely obscured in the visible range, was still clearly visible in the reconstructed holographic image (Fig. 1(g)). These tests demonstrate the capability of THz DH to work in scattering configuration, even through visible opaque media. The remarkable robustness of this technique to vibrations is a key requirement for outdoor operation and opens interesting perspectives for its deployment in industrial and homeland security applications.

**Figure 2:**
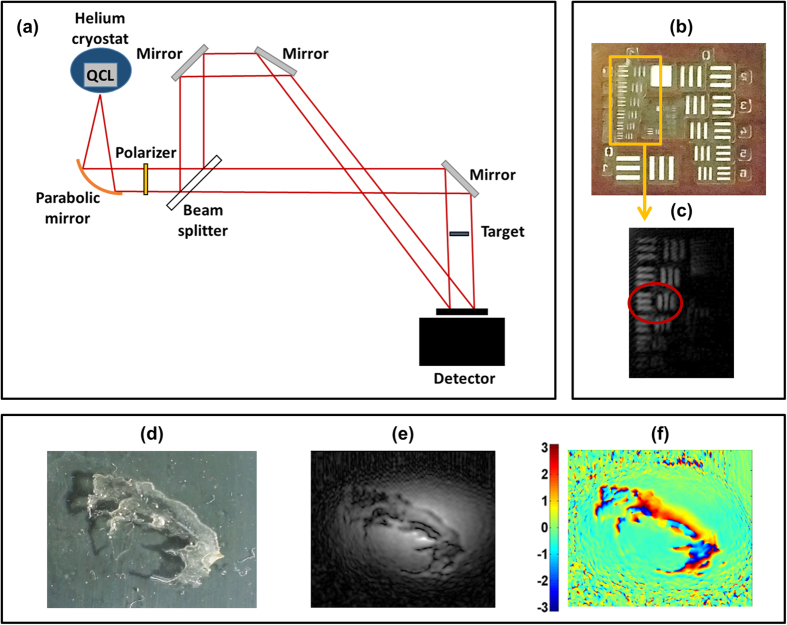
THz transmission DH. (**a**) Sketch of the experimental setup. **(b)** 1951 USAF resolution test chart R 74 (group 1 inside the yellow square). (**c**) Wavefront amplitude reconstruction of the lines belonging to group 1 (in the red circle, element 3 lines, with a thickness of 227 μm). **(d)** Picture of a 30 μm thick human skin histological slice. (**e**,**f**) Reconstructed amplitude (**e**) and phase (**f**) of the transmitted THz wavefront.

The aforementioned capability of THz radiation to penetrate several materials was exploited in experiments of transmission holography. In this case a flat mirror redirected the object beam through the target before impinging on the camera, while the target plane has been kept orthogonal to the THz beam (Fig. 2(a)). At first, we investigated the spatial resolution of such imaging scheme using an USAF resolution test chart (Fig. 2(b)), kept at 2 cm from the detector. As shown in Fig. 2(c), we were able to resolve patterns as small as 200 μm, in both x and y directions. This value, slightly higher than the intrinsic resolution limit^26^ (λd/(N_x_d_p_) × λd/(N_y_d_p_) = 133 μm × 178 μm, where N_x_, N_y_ are the array dimensions in pixels), could be enhanced by reducing the reference wavefront aberrations via spatial filtering. It is worth noting that the resolution depends on the distance d between the object and the camera. This can make THz holography competitive with visible/near-IR holography whenever large objects are investigated. In this case, the larger field of view resulting from the use of THz radiation allows reducing the distance between the object and the camera and somehow compensating the resolution loss resulting from the use of a longer wavelength.

THz imaging is of special interest for biology, due to the non-ionizing nature of THz waves. Accordingly, high resolution amplitude (Fig. 2(e)) and phase (Fig. 2(f)) images of a 30 μm thick histological blank slice of healthy human skin (Fig. 2(d)) were extracted from the relative holograms. In the THz amplitude image, epidermis and dermis skin layers can be distinguished, similarly to the visible microscope image, while, from the phase image, complementary information about the optical path length through the sample can be extracted. The assessed potential of 2D THz amplitude and phase imaging of biological samples can find wide application in histopathology, since high absorption of THz radiation in water is expected to provide a valuable tool for cancer diagnosis due to the abnormal hydration of tumour tissues^27^,^28^,^29^. The real-time capability of digital holography can help limit the impact of dehydration of freshly excised tissues^28^ and is well suited for in-vivo diagnostic of skin cancers.

**Figure 3:**
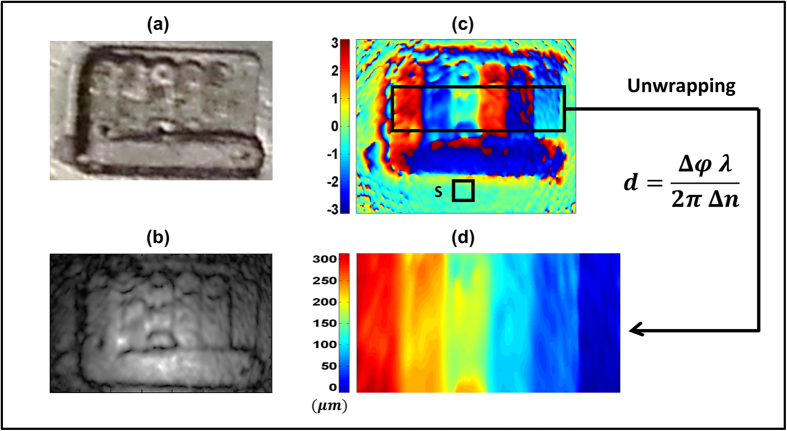
THz optical path length measurement. (**a**) Picture of the measured black polypropylene sheet with six, 1 mm wide, grooves of depth increasing by 50 μm steps. (**b**,**c**) Reconstructed amplitude (**b**) and phase (**c**) of the transmitted THz wavefront. S is the flat portion of the sheet considered for the calculation of the vertical resolution limit. (**d**) Particular of the wavefront phase reconstruction after phase unwrapping and rescaling from phase to depth.

In a further experiment, we demonstrate the capability of our system to measure optical path lengths through plastic materials. To this aim, a 1-mm thick black polypropylene sheet was processed on purpose, by etching six grooves of increasing depths on its surface (Fig. 3(a)). In this case, both amplitude (Fig. 3(b)) and phase images of the sample were retrieved. In order to evaluate the depth d of the grooves, a phase image of the sample was subtracted from a reference phase image of an un-etched portion of the plastic sheet and the resulting phase Δφ (Fig. 3(c)) was unwrapped and rescaled (Fig. 3(d)) into: d = Δφ λ/(2π Δn), where Δn is the difference between the polypropylene and air refractive indices. With this technique it is possible to measure path length variations equal to λ or larger, depending on the phase unwrapping capability. The achieved resolution, inferred from the standard deviation of the depth values over a flat area (Fig. 3(d)), approached ≈4 μm, limited by the low power and quality of our THz beam. Although this value is quite higher than the state of the art for DH, <λ/100^30^, it is still an order of magnitude lower than the typical axial resolution of THz tomography, that is limited by the THz pulse length^31^. The provided performances, combined with the real-time 2D acquisition capability, make THz DH a useful tool for the measurement of thickness of plastic sheets and, in general, for in-line non-destructive testing of dielectric objects in industrial processes.

## Conclusions

In this letter we have reported on real-time scattering and transmission THz DH, pointing out the remarkable potential of this technique through the realization of high quality holograms of several samples. The wide field of view and the extremely low sensitivity to vibrations, combined with the capability of penetrating various materials, make THz DH a promising tool for both security and industrial applications (e.g., non-ionizing body scanning, packaging inspection systems, plastic thickness evaluation, etc.) as well as for bio-photonics and cultural heritage investigations. DH in the THz range has been shown to possess unique features allowing new unusual applications, as compared to DH in other spectral regions.

## Methods

### Experimental Setup

The coherent source used for all the DH acquisitions is a 2.8 THz QCL (grown by a commercial provider) based on a bound-to-continuum active region, included in a 150 μm wide single-metal ridge waveguide cleaved to form a 2 mm Fabry Perot cavity^32^. The QCL was kept at 20 K in a liquid He flux cryostat and was driven with a CW injected current set at 750 mA, so as to obtain single mode operation and an output power ≈4 mW. Holograms were recorded by means of a room-temperature microbolometric thermocamera, Miricle 307 k (by Thermoteknix). The camera focal plane array, composed by 640 × 480 amorphous Si micro-bolometers with a pixel pitch of 25 μm × 25 μm, operates at a frame rate of 25 s^−1^. The detector is designed for the 8–12 μm spectral range, but exhibits high sensitivity also at 2.8 THz, as confirmed by our experiments.

As shown in Figs 1(a) and 2(a), the QCL beam, linearly polarized along the horizontal axis, was collimated using a f1 gold off axis parabolic mirror in order to obtain a 1.5 cm diameter beam, which was then split into reference and scattered/transmitted object beams by means of a Mylar beam splitter. The relative intensity of the two beams was tuned by means of a THz wire grid polarizer positioned before the beam splitter, in order to obtain high contrast fringes. The 50 Lire coin, the patterned polypropylene sheet and the human skin sample are all ≈1.5 cm wide; the inscription on the metallic plate is 7.5 cm long and the letters are 4 mm high. The targets were positioned at a distance from the detector ≈2 cm, in the case of transmission hologram acquisition, and ≈3.5 cm for the acquisition of scattering holograms. In the latter case, due to the ≈π/4 angle of the targets with respect to the thermocamera, the reconstructed image is shrunk by a factor √2/2 in the horizontal direction. The rigorous procedure for reconstructing the image of a planar object tilted with respect to the hologram plane has been recently addressed by P. Zolliker and E. Hack^33^.

### Real time hologram reconstruction

In DH, the reconstruction of the object beam is numerically performed by modelling the diffraction of a reconstruction beam (equal to the reference beam used for the acquisition) impinging on the recorded digital hologram^16^, with the Fresnel-Kirchhoff integral:


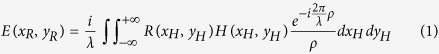


where *H*(*x*_*H*_, *y*_*H*_) and *R*(*x*_*H*_, *y*_*H*_) are the recorded hologram and the reconstruction beam wavefront in the hologram plane, *E*(*x*_*R*_, *y*_*R*_) is the scattered/transmitted object wavefront in the reconstruction plane and 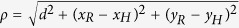 is the distance between points belonging to these two planes. For small propagation angles, the above integral can be simplified into the Fresnel diffraction integral, which can be calculated as a (discrete) Fourier Transform (DFT)^16^:


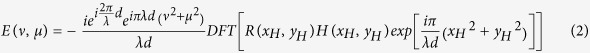


with *v* = *x*_H_/*λd* and *μ* = *y*_*H*_/*λd*.

To process our THz holograms we wrote a Matlab routine which efficiently computes the above DFT by means of a Fast Fourier Transform (FFT) algorithm and returns the amplitude and phase of the scattered/transmitted object wavefront, at a maximum frame rate of 5 Hz. In addition, the routine can calculate phase difference images with respect to a reference phase image. The routine includes a few manipulations, aimed at improving the quality of the reconstructed images: i) suppression of the zero diffraction order by means of one of three different numerical filters applied to the acquired holograms (subtraction of the average intensity, high pass filter, band pass filter); ii) speckle noise reduction; iii) hologram processing with a Gaussian apodization mask, aimed at reducing the low frequency noise arising from Fourier transformation due to the finite detector dimensions; iv) zero padding procedure^34^, which improves the final image resolution by including the recorded hologram into a frame filled with zeroes, so as to artificially increase its dimension.

## Additional Information

**How to cite this article**: Locatelli, M. *et al.* Real-time terahertz digital holography with a quantum cascade laser. *Sci. Rep.* **5**, 13566; doi: 10.1038/srep13566 (2015).

## Supplementary Material

Supplementary video
